# The (+)-Brevipolide H Displays Anticancer Activity against Human Castration-Resistant Prostate Cancer: The Role of Oxidative Stress and Akt/mTOR/p70S6K-Dependent Pathways in G1 Checkpoint Arrest and Apoptosis

**DOI:** 10.3390/molecules25122929

**Published:** 2020-06-25

**Authors:** Yi-Hua Sheng, Wohn-Jenn Leu, Ching-Nung Chen, Jui-Ling Hsu, Ying-Tung Liu, Lih-Ching Hsu, Duen-Ren Hou, Jih-Hwa Guh

**Affiliations:** 1 School of Pharmacy, National Taiwan University, No.33, Linsen S. Rd., Zhongzheng Dist., Taipei 100, Taiwan; yihua.sheng@rutgers.edu (Y.-H.S.); r00423018@gmail.com (W.-J.L.); d97423004@ntu.edu.tw (J.-L.H.); r05423012@ntu.edu.tw (Y.-T.L.); lhsu@ntu.edu.tw (L.-C.H.); 2 Department of Chemistry, National Central University, No. 300 Jhong-Da Road, Jhong-li, Taoyuan 32001, Taiwan; kjlncuuhhh@gmail.com; 3 Department of Pharmacy, New Taipei Municipal TuCheng Hospital, Chang Gung Memorial Hospital, New Taipei City 236, Taiwan

**Keywords:** (+)-brevipolide H, G1 checkpoint arrest, oxidative stress, Akt/mTOR/p70S6K-dependent pathway, mitochondrial dysfunction

## Abstract

Because conventional chemotherapy is not sufficiently effective against prostate cancer, various examinations have been performed to identify anticancer activity of naturally occurring components and their mechanisms of action. The (+)-brevipolide H, an α-pyrone-based natural compound, induced potent and long-term anticancer effects in human castration-resistant prostate cancer (CRPC) PC-3 cells. Flow cytofluorometric analysis with propidium iodide staining showed (+)-brevipolide H-induced G1 arrest of cell cycle and subsequent apoptosis through induction of caspase cascades. Since Akt/mTOR pathway has been well substantiated in participating in cell cycle progression in G1 phase, its signaling and downstream regulators were examined. Consequently, (+)-brevipolide H inhibited the signaling pathway of Akt/mTOR/p70S6K. The c-Myc inhibition and downregulation of G1 phase cyclins were also attributed to (+)-brevipolide H action. Overexpression of myristoylated Akt significantly rescued mTOR/p70S6K and downstream signaling under (+)-brevipolide H treatment. ROS and Ca^2+^, two key mediators in regulating intracellular signaling, were determined, showing that (+)-brevipolide H interactively induced ROS production and an increase of intracellular Ca^2+^ levels. The (+)-Brevipolide H also induced the downregulation of anti-apoptotic Bcl-2 family proteins (Bcl-2 and Bcl-xL) and loss of mitochondrial membrane potential, indicating the contribution of mitochondrial dysfunction to apoptosis. In conclusion, the data suggest that (+)-brevipolide H displays anticancer activity through crosstalk between ROS production and intracellular Ca^2+^ mobilization. In addition, suppression of Akt/mTOR/p70S6K pathway associated with downregulation of G1 phase cyclins contributes to (+)-brevipolide H-mediated anticancer activity, which ultimately causes mitochondrial dysfunction and cell apoptosis. The data also support the biological significance and, possibly, clinically important development of natural product-based anticancer approaches.

## 1. Introduction

Prostate cancer is one of the most common malignancies in elderly men and is a leading cause of cancer deaths globally. Death occurs in the later stages of prostate cancer progression in metastatic hormone-sensitive and majorly in castration-resistant prostate cancer (CRPC) [1]. Management of CRPC usually involves treatments targeting the androgen receptor pathway, chemotherapy, radiotherapy, and other approaches (i.e., immunotherapy). Nevertheless, most patients with CRPC eventually surrender to the disease. Better treatments are, therefore, required. The phosphoinositide 3-kinase (PI3K)/Akt (protein kinase B)/mammalian target of rapamycin (mTOR) signaling axis is a crucial signaling pathway in both physiological and pathological conditions, and has been evidenced to interact with a variety of pathways, such as mitogen-activated protein kinases (MAPKs), hypoxia-inducible factor, Ras, Notch and Wnt/β-catenin, which are responsible for cell proliferation, survival, and drug resistance [2,3,4,5]. Accordingly, PI3K/Akt/mTOR signaling is a core pathway responsible for tumor progression and drug resistance and appears to be a potential target in various cancer treatments, including CRPC [6,7,8,9].

Natural product-based drug research and development have drawn considerable interest, particularly in the area of cancer. It has been reported that about 50% of the approved anticancer small molecules in recent decades are either natural products or directly derived therefrom [10]. The utilization of natural products and their structures in the research and development of cancer drug entities continues to gain substantial attention. Brevipolides, a series of 5,6-dihydro-α-pyrone derivatives from *Hyptis brevipes*, have been found to exhibit anticancer activities against colon cancer, breast cancer, nasopharyngeal cancer, laryngeal cancer, and prostate cancer [11,12,13]. However, the anticancer mechanism of brevipolides has not been clearly identified, although several studies have reported that they display inhibitory activity on chemokine receptor CCR5 [14] and nuclear factor-κB (NF-κB) [11] using calcium mobilization and enzyme-based NF-κB assays. We successfully synthesized (+)-brevipolide H, one of the active components in brevipolides, and the bioactive examination showed potent anti-proliferative effects in CRPC cell lines [13]. The results prompted the elucidation of the anti-CRPC mechanism of (+)-brevipolide H in this study using the PC-3 cell model, a bone metastasis-derived CRPC cell line. Furthermore, PC-3 is a *phosphatase and tensin homologue* (*PTEN*) null cell line in which the tumor suppressor PTEN expression is absent. Because PTEN is specifically identified as a negative regulator of the PI3K signaling, the absence of PTEN expression enables high activity of PI3K/Akt signaling in PC-3 cells [15].

In this study, (+)-brevipolide H-mediated anti-CRPC mechanism was elucidated in light of the crosstalk between intracellular calcium mobilization and oxidative stress in the regulation of PI3K/Akt/mTOR signaling and cellular events, including cell cycle perturbation, anti-proliferation, mitochondrial stress, and cell apoptosis. To the best of our knowledge, this is the first report studying ROS and calcium interactively regulating PI3K/Akt/mTOR signaling pathways to brevipolide action.

## 2. Results

### 2.1. The (+)-Brevipolide H Induces Anti-Proliferative Effect in PC-3 Cells with Long-Term Efficacy

**Figure 1:**
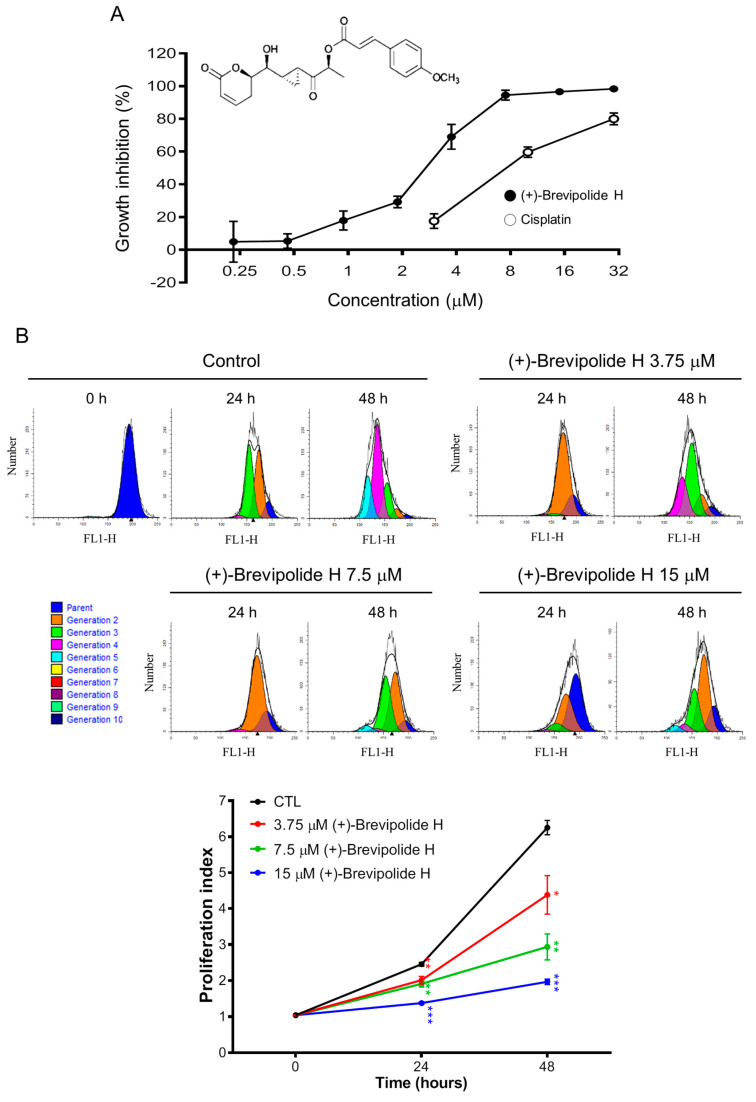
Effect of (+)-brevipolide H on cell proliferation in PC-3 cells. (**A**) PC-3 cells were incubated in the absence or presence of (+)-brevipolide H for 96 h. After treatment, the cells were fixed and stained for SRB assay. (**B**) The cells were stained with CFSE and incubated in the absence or presence of (+)-brevipolide H for 24 or 48 h. Cell proliferation was detected using FACScan flow cytometer. Proliferation index was calculated by using ModFit LT^TM^ 3.3 to quantify the inhibition of cell proliferation. Data are expressed as mean ± SEM of three independent experiments; * *p* < 0.05, ** *p* < 0.01, and *** *p* < 0.001 compared with the respective control.

The sulforhodamine B (SRB) assay, which relies on the detection of SRB binding to protein basic amino acid residues of trichloroacetic acid-fixed cells, is one of the most widely used methods for anti-proliferative examination. As a result, (+)-brevipolide H displayed concentration-dependent inhibition of PC-3 cell proliferation with the half maximal inhibitory concentration (IC_50_) of 2.72 µM (Figure 1A). Flow cytometric analysis of CFSE staining was used to substantiate anti-proliferative effects, since CFSE fluorescence in daughter cells is half that of the parent when cells divide. The data demonstrated that the proliferation index in the 48-h control group was 6.25 ± 0.20, but decreased to 4.38 ± 0.53, 2.94 ± 0.36, and 1.97 ± 0.08 when exposed to (+)-brevipolide H at 3.75, 7.5, and 15 µM, respectively (Figure 1B). The data verified the anti-proliferative effect of (+)-brevipolide H action. Moreover, the long-term (seven days) effects of (+)-brevipolide H on anchorage-dependent growth of PC-3 cells were examined using clonogenic assay. The (+)-brevipolide H resulted in a concentration-dependent inhibition of colony formation with an IC_50_ of 0.37 ± 0.06 µM (Supplementary Figure S1), suggesting long-term efficacy of (+)-brevipolide H.

### 2.2. The (+)-Brevipolide H Induces G1 Arrest of the Cell Cycle and Downregulates G1 Cyclins

**Figure 2:**
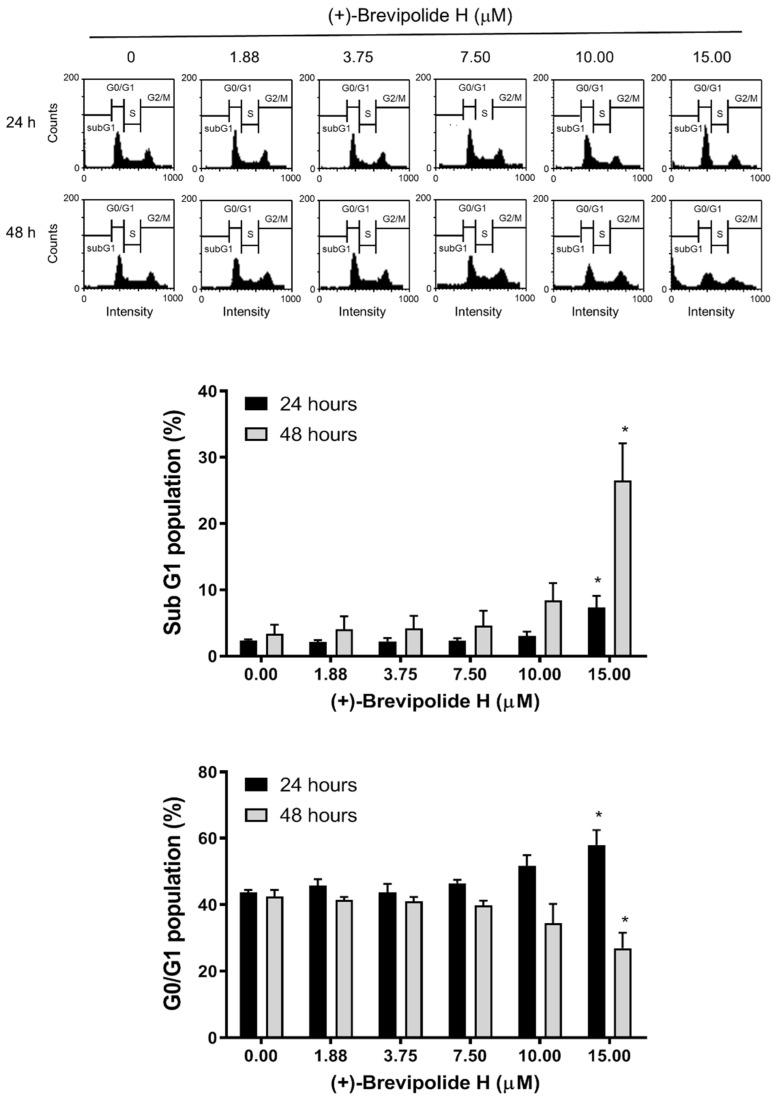
Effect of (+)-brevipolide H on cell cycle distribution in PC-3 cells. The cells were incubated in the absence or presence of (+)-brevipolide H for 24 or 48 h and then stained with propidium iodide. The distribution of cell population was examined using FACScan flow cytometric analysis. Quantitative data of cell population at both apoptotic sub-G1 and G0/G1 were analyzed with BD CellQuest^TM^ Pro Software. Data are expressed as mean ± SEM of three independent experiments; * *p* < 0.05 compared with the respective control.

**Figure 3:**
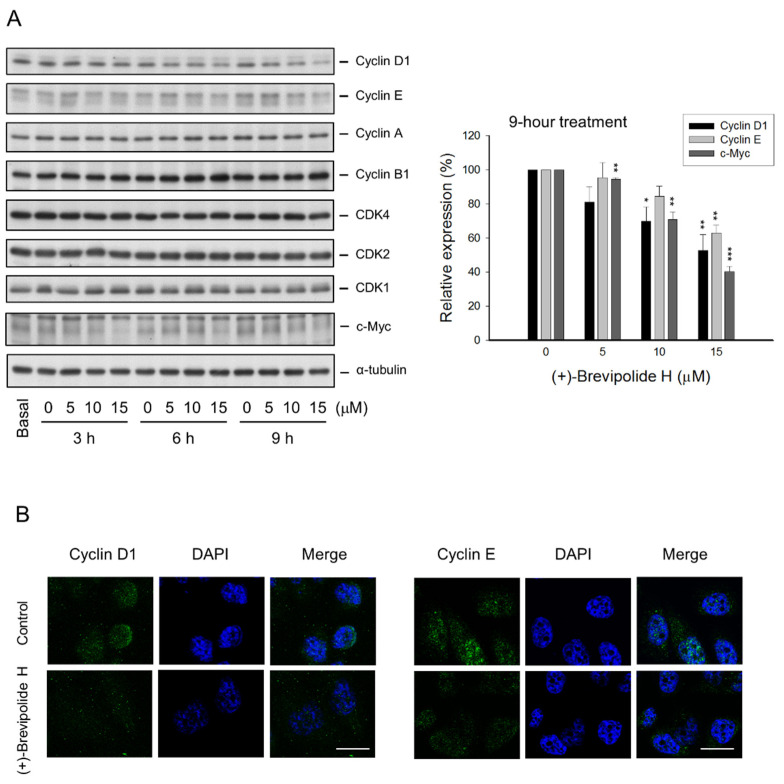
Effect of (+)-brevipolide H on the expression of cell cycle related proteins. (**A**) PC-3 cells were incubated in the absence or presence of (+)-brevipolide H for the indicated concentrations and times. The cells were then collected and lysed for the detection of protein expression by Western blot analysis. Quantitative data of the relative expression under 9-h treatment are expressed as mean ± SEM of three independent experiments using Bio-Rad Image Lab^TM^ Software (Bio-Rad, Hercules, CA, USA). The * *p* < 0.05, ** *p* < 0.01, and *** *p* < 0.001 compared with the control. (**B**) PC-3 cells were starved at fetal bovine serum (FBS)-free medium for 24 h and then incubated in FBS-containing (10%) medium in the absence or presence of (+)-brevipolide H (15 µM) for 3 h. The confocal immunofluorescence microscopic examination was performed. *Bar*, 20 µm.

The G1 checkpoint, also known as the restriction point, is the phase at which the cell is committed to entering the cell cycle and is vulnerable to various cellular stresses to arrest [16]. The (+)-brevipolide H in 24-h treatment induced a concentration-dependent increase of G1 phase population and a subsequent increase of apoptosis after a 48-h exposure (Figure 2), suggesting an induction of an apoptotic G1 arrest effect. Cell apoptosis was further substantiated by the activation of caspase cascades (Supplementary Figure S2). Cyclin D1/cyclin-dependent kinase (CDK) 4 and cyclin E/CDK2 complexes are key kinase complexes in the G1 phase. Both complexes promote G1 phase progression by inhibiting retinoblastoma protein (Rb) through phosphorylation. Hyper-phosphorylated Rb will no longer interact with E2F transcription factor, allowing it to transcribe genes necessary for S phase entry [17,18]. The c-Myc, a critical oncoprotein which collaborates with various growth factors, Ras, and PI3K/Akt through coordination in regulating both cyclin D1 and cyclin E expressions, is implicated in enhancing tumor formation and driving aggressiveness of tumors [19,20]. The data in Figure 3A showed that (+)-brevipolide H induced a significant downregulation of protein expressions of both cyclin D1 and cyclin E, and their upstream regulator c-Myc. The data were correlated with the induction of the G1 arrest of the cell cycle. Cyclin D1 is synthesized rapidly and accumulates in the nucleus during early G1 phase and is degraded when entering the S phase; in contrast, cyclin E is required for the G1/S transition [21,22]. The confocal imaging in Figure 3B demonstrates a greater level of cyclin E in the nucleus than that of cyclin D1; furthermore, (+)-brevipolide H displayed profound inhibition of both protein expressions. The data suggested the inhibitory effect of (+)-brevipolide H on the late G1 phase.

### 2.3. The (+)-Brevipolide H Inhibits Akt/mTOR/p70S6K Signaling Pathways

**Figure 4:**
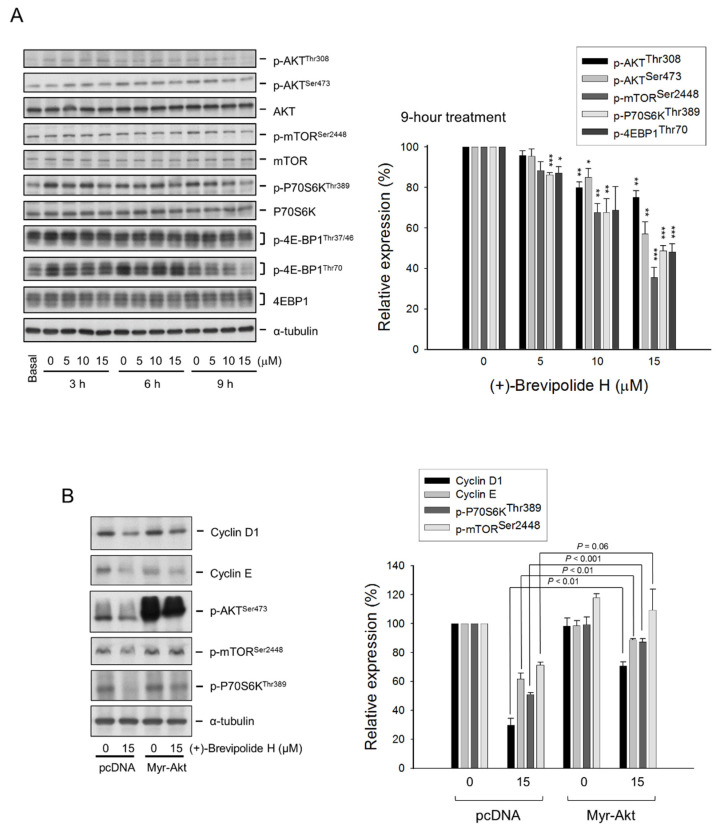
Effect of (+)-brevipolide H on the expressions of Akt/mTOR/p70S6K pathway proteins. (**A**) PC-3 cells were incubated in the absence or presence of (+)-brevipolide H for the indicated concentrations and times. The cells were then collected and lysed for the detection of protein expression by Western blot analysis. Quantitative data under the 9-h treatment are expressed as mean ± SEM of three independent experiments using Bio-Rad Image Lab^TM^ Software; * *p* < 0.05, ** *p* < 0.01, and *** *p* < 0.001 compared with the control. (**B**) PC-3 cells were transfected with control vector or Myr-Akt and treated without or with 15 µM (+)-brevipolide H for 9 h. The protein expression was examined using Western blot analysis. Quantitative data of the relative protein expression were analyzed using Bio-Rad Image Lab^TM^ Software.

During the G1 phase, the cell synthesizes mRNA and proteins necessary for DNA synthesis. Once the required cellular processes are complete, the cell enters the S phase. PI3K/Akt signaling activity is required for cell growth and cell cycle entry. Moreover, it was evident that PI3K activity is essential at the late G1 phase for the entry into the S phase [23]. The mTOR, which belongs to the PI3K kinase-related kinase superfamily, plays a key role in translational control, which is crucial in regulating cell proliferation, cell growth, and survival. The eukaryotic translation initiation factor 4E-binding protein 1 (4E-BP1), a translation repressor protein, interacts with eukaryotic translation initiation factor 4E (eIF4E), which recruits 40S ribosomal subunits to the 5′ end of mRNAs. The interaction between 4E-BP1 and eIF4E blocks complex association and suppresses translation [24,25]. Hyper-phosphorylation of 4E-BP1 disturbs this interaction, resulting in the activation of cap-dependent translation [25]. Notably, the phosphorylation of 4E-BP1 at threonine (Thr)37/Thr46 does not inhibit this interaction but primes 4E-BP1 for subsequent phosphorylation at serine (Ser) 65 and Thr70 [26]. The (+)-brevipolide H induced an inhibitory effect on p-Akt at both Thr308 and Ser473 residues, p-mTOR at Ser2448, p-p70S6K at Thr389, and p-4E-BP1 at Thr70, indicating the inhibition of the Akt/mTOR/p70S6K signaling pathway (Figure 4A).

Although activation of the Akt-dependent mTOR pathway is usually present in prostate cancer, it has been reported that mTOR activation is also dependent on kinases other than Akt [27]. To examine these possibilities, we tested the ability of myristoylated Akt (Myr-Akt), a constitutively active form of Akt, on the effect of several cellular targets. The data demonstrated that overexpression of Myr-Akt significantly rescued the (+)-brevipolide H-suppressed mTOR and p70S6K activities (Figure 4B), indicating an Akt-dependent mTOR/p70S6K signaling pathway. Notably, overexpression of Myr-Akt also profoundly prevented the downregulation of both cyclin D1 and cyclin E expressions (Figure 4B). The data supported a crucial role of Akt-dependent control of (+)-brevipolide H action in both cyclin D1 and cyclin E expressions.

### 2.4. Crosstalk between ROS and Calcium Plays a Key Role in (+)-Brevipolide H-Mediated Signaling Pathways

**Figure 5:**
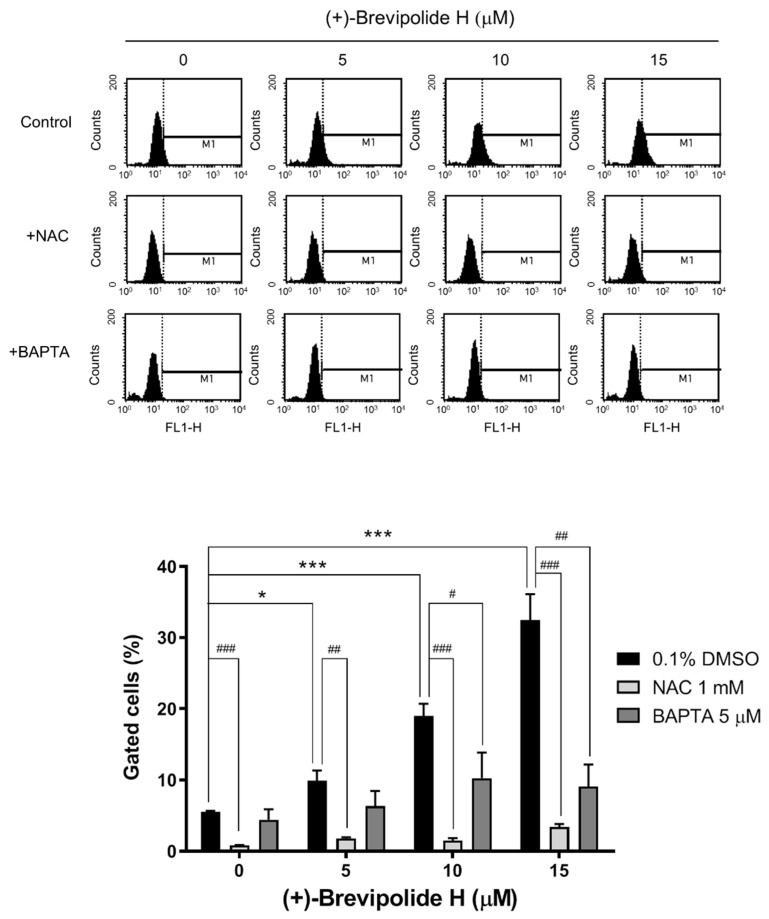
Effect of (+)-brevipolide H on ROS production in PC-3 cells. The cells were treated in the absence or presence of (+)-brevipolide H (5 to 15 µM), NAC (1 mM), and BAPTA (5 µM) for 1 h. ROS production was detected using FACScan flow cytometric analysis. Quantitative data of ROS generation were analyzed using BD CellQuest^TM^ Pro Software. Data are expressed as mean ± SEM of three independent experiments; * *p* < 0.05 and *** *p* < 0.001 compared with (+)-brevipolide H-free control and ^#^ *p* < 0.05, ^##^ *p* < 0.01 and ^###^ *p* < 0.001 compared with respective (+)-brevipolide H group.

ROS, in particular hydrogen peroxide, functions as a second messenger in signaling cascades which orchestrate cell survival, proliferation, and cell death [28,29]. Most importantly, ROS-sensitive signaling pathways, including MAPK, PI3K/Akt, and NF-κB pathways, are continually high in many types of cancers [30]. The data showed that (+)-brevipolide H induced a dramatic increase of NAC-inhibitable ROS production. (Figure 5). Moreover, NAC almost completely abolished (+)-brevipolide H-induced increase of G1 population and apoptosis (Supplementary Figure S3).

**Figure 6:**
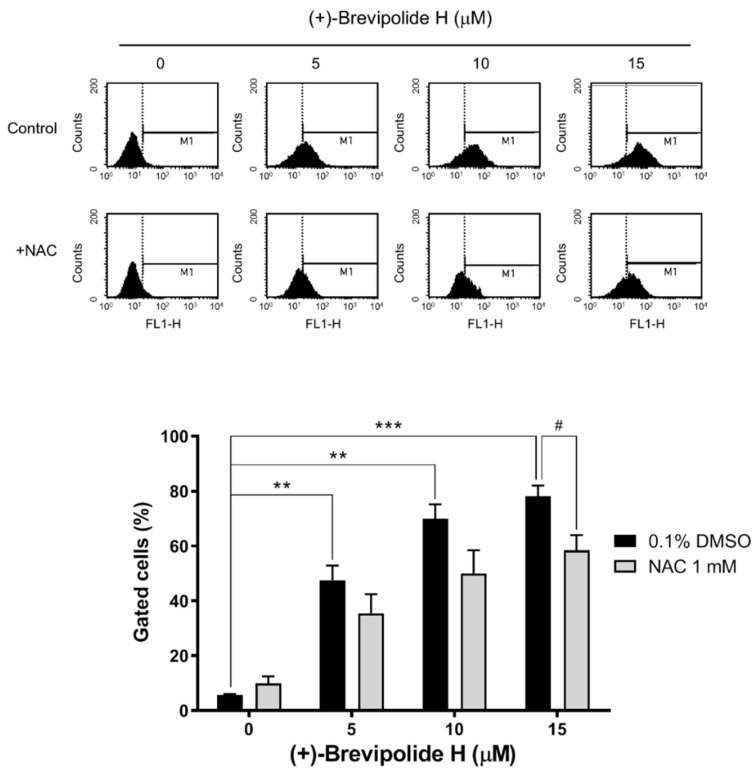
Effect of (+)-brevipolide H and NAC on the intracellular Ca^2+^ levels in PC-3 cells. The cells were incubated in the absence or presence of (+)-brevipolide H (5 to 15 µM) and NAC (1 mM) for 1 h. Intracellular Ca^2+^ levels were detected using FACScan flow cytometric analysis. Quantitative data were analyzed using BD CellQuest^TM^ Pro Software. Data are expressed as mean ± SEM of three independent experiments; ** *p* < 0.01 and *** *p* < 0.001 compared with (+)-brevipolide H-free control and ^#^ *p* < 0.05 compared with respective (+)-brevipolide H group.

Ca^2+^ is another ubiquitous second messenger involved in a variety of cellular functions, including cell cycle progression, cell survival, migration, and apoptosis [31]. It is noteworthy that the nature of the calcium signal is changed in cancer and with tumor progression and, therefore, various studies have suggested specific Ca^2+^ channels and pumps as drug targets for numerous cancer types, including prostate [32,33]. In this study, (+)-brevipolide H caused a substantial increase of intracellular Ca^2+^ levels, which was partly but significantly inhibited by NAC (Figure 6). Together with data showing that the intracellular calcium chelator BAPTA significantly diminished ROS generation to (+)-brevipolide H action (Figure 5), it revealed that ROS, in concert with intracellular Ca^2+^, served as a key messenger in regulating (+)-brevipolide H action on G1 arrest and apoptosis function. Notably, the regulation of intracellular Ca^2+^ content involves both Ca^2+^ entry from extracellular spaces and Ca^2+^ release from intracellular stores. Therefore, the (+)-brevipolide H-induced effect was examined in the presence (0.4 mM Ca^2+^ solution) or absence [Ca^2+^-free/ethylene glycol tetraacetic acid (EGTA) solution] of extracellular Ca^2+^. As a consequence, both conditions allowed a concentration-dependent increase of cytosolic Ca^2+^ levels under (+)-brevipolide H treatment (Supplementary Figure S4), suggesting the involvement of both extracellular Ca^2+^ entry and intracellular Ca^2+^ release.

### 2.5. The (+)-Brevipolide H Induced Downregulation of Anti-Apoptotic Bcl-2 Family Proteins and Loss of Mitochondrial Membrane Potential

**Figure 7:**
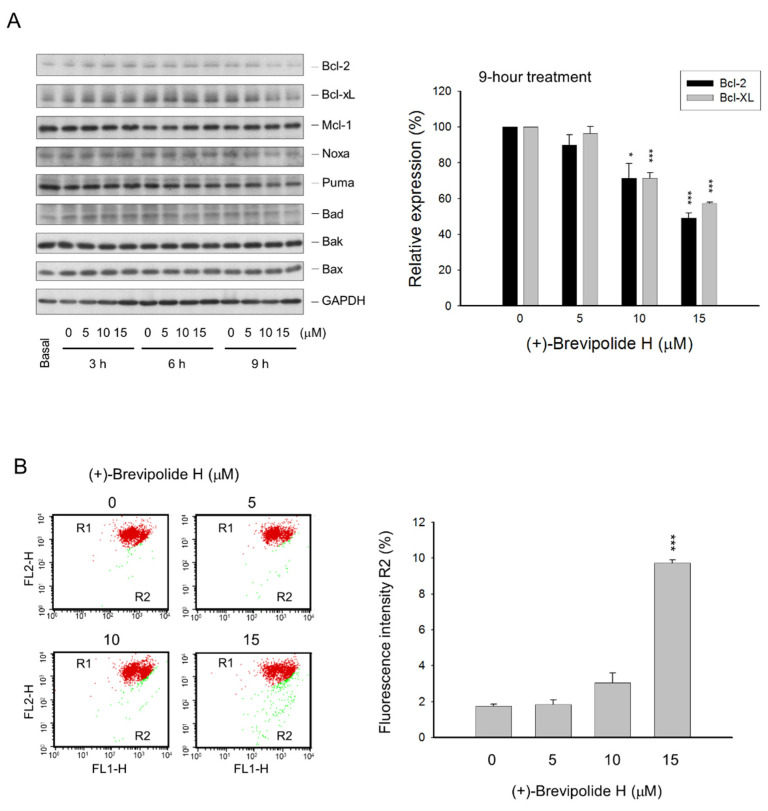
Effect of (+)-brevipolide H on the expressions of Bcl-2 family of proteins and mitochondrial membrane potential in PC-3 cells. (**A**) The cells were incubated in the absence or presence of (+)-brevipolide H for the indicated concentrations and times. The cells were then collected and lysed for the detection of protein expression by Western blot analysis. Quantitative data under the 9-h treatment are expressed as mean ± SEM of three independent experiments using Bio-Rad Image Lab^TM^ Software; * *p* < 0.05 and *** *p* < 0.001 compared with the respective control. (**B**) The cells were incubated in the absence or presence of (+)-brevipolide H for 24 h and then stained with JC-1 to monitor mitochondrial membrane potential. Quantitative data were analyzed using BD CellQuest^TM^ Pro Software and expressed as mean ± SEM of three independent experiments; *** *p* < 0.001 compared with the control.

Mitochondria are at the center of the cellular energy metabolism, being the major source for ATP production. The mitochondrial function is critically regulated by Ca^2+^. Overload of mitochondrial matrix Ca^2+^ leads to augmented production of ROS, inducing the permeability transition pore and cytochrome c release, which ultimately result in apoptosis [34,35]. The effect of (+)-brevipolide H on mitochondrial function was determined. The (+)-brevipolide H induced a profound downregulation of B-cell lymphoma 2 (Bcl-2) and Bcl-extra large (Bcl-xL), two pro-survival Bcl-2 families of proteins, but not the other family members (Figure 7A). Furthermore, (+)-brevipolide H resulted in a profound loss of mitochondrial membrane potential (Figure 7B). The data suggested the mitochondrial dysfunction in cells in response to (+)-brevipolide H.

## 3. Discussion

Discovery and development of new drugs, in particular anticancer drugs, from natural products, has been the focus of much research. The α-pyrone is one of the most abundant scaffolds in naturally occurring molecules, in which many are cytotoxic agents with anticancer potentials, such as bufadienolides, gibepyrones, higginsianins, and pironetin [36,37,38,39,40]. The (+)-brevipolide H, which belongs to the naturally occurring 5,6-dihydro-α-pyrone derivatives, displayed anti-proliferative and apoptotic activities against CRPCs in the present work. The (+)-brevipolide H is structurally related to pironectin, which is a crystallographically verified compound targeting α-tubulin and inducing apoptosis in cancer cells through the induction of microtubule disassembly and mitotic arrest of the cell cycle [41]. To elucidate the mechanism in PC-3 cells under (+)-brevipolide H exposure, the distribution of cell cycle phases was examined showing the arrest of G1 other than the mitotic phase. Further substantiation also demonstrated the downregulation of protein expressions in G1 cyclins—cyclin D1 and cyclin E—and suppression of their nuclear localization. The result was distinguishable from that of cyclin B1 upregulation under mitotic arrest stress induced by anti-tubulin agents [42,43].

The mitochondrion is a critical gatekeeper of life and death in the cell and is one of the most susceptible organelles responsive to apoptotic stimuli through the disruption of ATP synthesis, generation of ROS, and release of proapoptotic proteins [34]. We demonstrated that the mitochondrion was damaged under (+)-brevipolide H exposure by detecting the downregulation of Bcl-2 and Bcl-xL, and the loss of mitochondrial membrane potential; the mitochondrial dysfunction would impair ATP synthesis. Cell proliferation has a high reliance on energy consumption that regulates the checkpoints of the cell cycle [44]. Numerous studies have suggested the entry of cells into the G1 phase involved a burst of mitochondrial activity since the enzyme activities required in oxidative phosphorylation are increased in the G1 phase [45]. These studies support (+)-brevipolide H-induced G1 arrest of the cell cycle and subsequent apoptosis.

ROS have been widely characterized as being not just detrimental by-products of mitochondrial respiration, but also as being crucial for cell signaling [34]. The primary ROS produced by mitochondria is superoxide (O2•^−^), which is converted to H_2_O_2_. Mitochondrial membrane potential, a driving force for ATP synthesis, is a crucial factor of ROS production. Depolarization below a certain potential may indicate damaged mitochondrial function and induce ROS production. In a feedback mechanism, ROS can further trigger mitochondrial uncoupling [46,47]. Our data showed that (+)-brevipolide H induced the loss of mitochondrial membrane potential (depolarization) in PC-3 cells and triggered ROS production. Furthermore, it has been reported that oxidative stress to the respiration chain can depolarize the mitochondrial inner membrane, disturbing Ca^2+^ reuptake. Furthermore, ROS can induce certain Ca^2+^ release pathways from mitochondria through the oxidation of some vicinal thiols [48]. The (+)-brevipolide H-induced increase of intracellular Ca^2+^ levels was suppressed by NAC, suggesting the contribution of oxidative stress to Ca^2+^ flux. On the other hand, it is evident that Ca^2+^ can also stimulate ROS production through several events, including an increase of electron flow into the respiration chain, inhibition of respiration at complex I and IV by produced nitric oxide, and inhibition of complex III by opening of the permeability transition pore and cytochrome c release [34,49]. Our data showed that BAPTA significantly diminished ROS generation to (+)-brevipolide H action, supporting the contribution of Ca^2+^ flux to oxidative stress. In addition to the mitochondrion, the endoplasmic reticulum (ER) is another important organelle in regulating ROS production and Ca^2+^ mobilization. Evidence abounds that during ER-involved stress conditions, various stimuli can induce ROS production through redox-signaling pathways and Ca^2+^ release from related channels, i.e., inositol trisphosphate (InsP3) and ryanodine receptors, and sarco/endoplasmic reticulum Ca^2+^-ATPase (SERCA) residing in the endomembrane [50]. It is worth noting that crosstalk exists between mitochondrion and ER where ROS and Ca^2+^ flux play crucial roles since various Ca^2+^-related proteins, including InsP3 receptor, SERCA pumps, and mitochondrial Ca^2+^ uniporter, are redox sensitive [51]. In this study, both extracellular Ca^2+^ entry and intracellular Ca^2+^ release were involved in (+)-brevipolide H-mediated signaling. Although current data could not distinguish the contribution to Ca^2+^ flux between these organelles, it was suggested that, at least, the mitochondrion was involved in this activity, although the role of ER remained unclear.

PI3K/Akt activation takes place at two distinct times during the progression of the cell cycle. The first occurs immediately after growth factor exposure and the second at late G1 phase since PI3K activation can stimulate cyclin E/Cdk2 activity and the entry of S phase for DNA synthesis, whereas rapamycin (the best known mTOR inhibitor) completely abolishes this activity [52,53]. Furthermore, blockade of late-G1 PI3K activity can induce an inhibitory effect on c-Myc expression; however, expression of a stable c-Myc mutant rescues the effect and restores the entry of the S phase, suggesting the requirement of c-Myc stabilization in PI3K-mediated induction of DNA synthesis [52]. Our data were in agreement with these studies, showing that (+)-brevipolide H suppressed Akt/mTOR pathway associated with the inhibition of c-Myc in stopping the cell cycle at the late G1 phase. One crucial issue is that Akt may interact with and phosphorylate InsP3 receptors to reduce the Ca^2+^ release capability in cells responsive to survival stimulus and to decrease cellular susceptibility under apoptotic stress through the pathway requiring decreased calcium mobilization in the crosstalk between the ER and the mitochondrion [54,55]. This might, at least partly, explain the suppression of Ca^2+^ flux under (+)-brevipolide H exposure.

The data in the present work revealed that both Bcl-2 and Bcl-xL were two Bcl-2 family members being downregulated to (+)-brevipolide H action. Bcl-2 has been shown to participate in carcinogenesis and development of androgen-independent prostate cancer. Upregulation of Bcl-2 expression has been reported to link the PI3K/Akt survival pathway [56]. Ren and colleagues reported that PI3K/Akt blockade in combination with Bcl-xL inhibition resulted in synergistic apoptosis, in particular in PTEN-mutant prostate cancer cells, whereas single PI3K/Akt inhibition did not [57]. The report suggests that the inhibition of Bcl-2 and Bcl-xL is efficient in combating with PI3K/Akt survival signaling. Our data supported this notion, since (+)-brevipolide H efficiently induced an anticancer effect in PTEN-null PC-3 cells through the inhibition of the PI3K/Akt pathway and downregulation of both Bcl-2 and Bcl-xL. Notably, Bcl-2 is not only associated with the outer mitochondrial membrane but also with the ER, and is able to decrease steady-state Ca^2+^ of the ER in an InsP3 receptor-dependent mechanism. Moreover, Bcl-2 overexpression reduces ER Ca^2+^ levels and, therefore, decreases InsP3 agonist-induced Ca^2+^ flux in the cytoplasm and in the mitochondria [55,58]. Similar Ca^2+^ flux modulation was observed by Bcl-xL [59]. These studies further supported the (+)-brevipolide H action of downregulating both Bcl-2 and Bcl-xL expressions, while increasing cytosolic Ca^2+^ levels through both intracellular store release and extracellular space influx.

**Figure 8:**
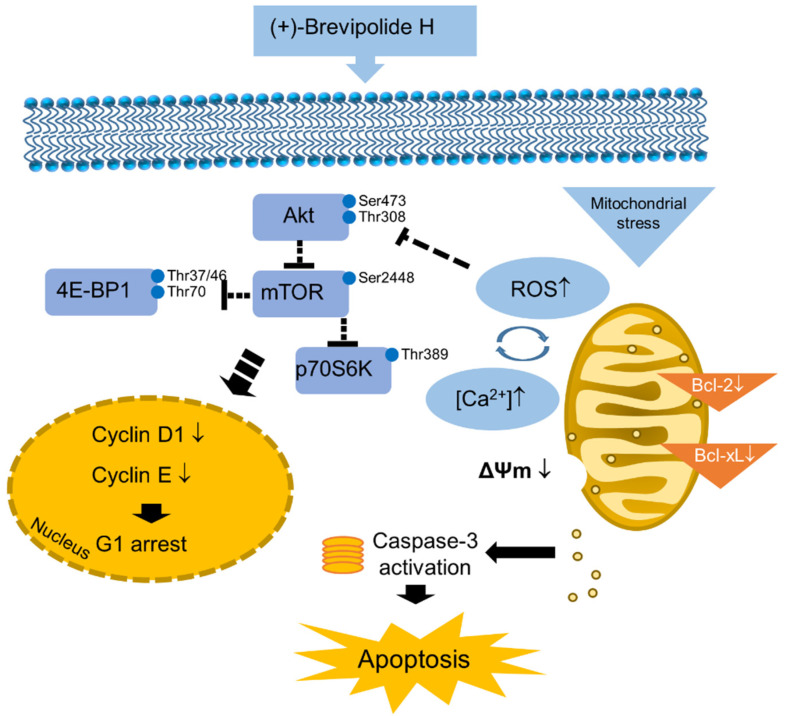
Signaling pathway of (+)-brevipolide H in PC-3 cells. The (+)-brevipolide H displays anticancer activity through mitochondrial stress in which the interplay between ROS production and increased cytosolic Ca^2+^ levels takes place. The inhibition of both Bcl-2 and Bcl-xL plays a crucial role on mitochondrial dysfunction. Akt/mTOR/p70S6K pathway was suppressed followed by the downregulation of cyclin D1 and cyclin E in (+)-brevipolide H-induced G1 checkpoint arrest. The cellular stress may ultimately induce apoptosis.

In conclusion, the data suggest that (+)-brevipolide H induces anticancer signaling in a sequential manner (Figure 8). It induces an interplay between ROS production and increased cytosolic Ca^2+^ levels, which are contributed to by both intracellular Ca^2+^ release and extracellular Ca^2+^ influx. Furthermore, the Akt/mTOR/p70S6K pathway was suppressed followed by the downregulation of cyclin D1 and cyclin E in (+)-brevipolide H-induced G1 checkpoint arrest. Notably, the inhibition of both Bcl-2 and Bcl-xL may play a crucial role, not only in mitochondrial dysfunction, but also in supporting intracellular calcium mobilization and Akt inhibition, although interactive regulation needs further elucidation.

## 4. Materials and Methods

### 4.1. Materials

Roswell Park Memorial Institute (RPMI) 1640 medium, Opti-Minimum Essential Medium (MEM), FBS, Pen-Strep-Ampho Solution (10,000 U/mL of penicillin, 10 mg/mL of streptomycin and 0.025 mg/mL of Amphotericin B), carboxyfluoresceinsuccinimidyl ester (CFSE), Lipofectamine 2000 transfection reagent, 5,5′,6,6′-tetrachloro-1,1′,3,3′-tetraethyl-benzimidazolylcarbocyanine iodide (JC-1) and 2′,7′-dichlorodihydrofluorescein diacetate (DCF-DA) were purchased from Thermo Fisher Scientific (Madison, WI, USA). Acetic acid, crystal violet, DL-dithiothreitol (DTT), propidium iodide (PI), phenylmethanesulfonyl fluoride (PMSF), EGTA, sodium fluoride (NaF), sodium orthovanadata (Na_3_VO_4_), N-acetyl-L-cysteine (NAC), and SRB were from Sigma Chemical (St. Louis, MO, USA). Antibody of caspase-3 was from Imgenex (San Diego, CA, USA). Antibodies of cleaved caspase-9, cyclin D1, p-4E-BP1^Thr70^, p-4E-BP1^Thr37/46^, 4E-BP1, p-Akt^Thr308^, p-Akt^Ser473^, Akt, p-p70S6K^Thr389^, p-mTOR^Ser2448^, and mTOR were obtained from Cell Signaling (Beverly, MA, USA). Antibodies of cyclin A, cyclin B1, cyclin E, CDK1, CDK2, CDK4, c-Myc, α-tubulin, Mcl-1, Bcl-2, Bcl-xL, Bak, Bad, Bax, Noxa, p53-upregulated modulator of apoptosis (Puma), glyceraldehyde 3-phosphate dehydrogenase (GAPDH), poly [ADP-ribose] polymerase-1 (PARP-1), horseradish peroxidase (HRP)-conjugated anti-mouse and anti-rabbit immunoglobulin G (IgG) were purchased from Santa Cruz Biotechnology (Santa Cruz, CA, USA). Antibody of p70S6K was from Abcam (Cambridge, MA, USA). Bio-Rad protein assay kit was from Bio-Rad (Hercules, CA, USA). Poly(vinylidene fluoride) (PVDF) membrane was purchased form Pall Gelman Laboratory (Ann Arbor, MI, USA). The (+)-brevipolide H was totally synthesized and published previously [13]. The compound was solubilized in DMSO. The final concentration of DMSO in cells was 0.1%.

### 4.2. Cell Culture

CRPC cell line PC-3, obtained from Bioresource Collection and Research Center (Hsinchu, Taiwan), was cultured in RPMI 1640 medium with 5% (*v*/*v*) FBS, 100 U/mL penicillin and 100 µg/mL streptomycin. Cultures were maintained in a 37 °C incubator with 5% CO_2_. Cells were detached by using 0.05% trypsin-EDTA for passaging at confluence.

### 4.3. Sulforhodamine B (SRB) Assay

PC-3 cells were cultured in 96-well plates in RPMI 1640 medium with 5% (*v*/*v*) FBS at a density of 3000 cells per well for 24 h. Cells of few wells were fixed with 10% trichloroacetic acid (TCA) to represent for cell numbers at the time of compound treatment (T_0_). Cells in control groups were incubated in 0.1% DMSO while cells in experimental groups were treated with the indicated compound for 96 h. After the treatment, cells were fixed with 10% TCA and stained with 0.4% (*w*/*v*) SRB dissolved in 1% acetic acid. Unbound dye was washed out with 1% acetic acid. The dye that bound to proteins was solubilized with 10 mM Tris base, and the absorbance of the solution was further measured at a wavelength of 515 nm to realize the inhibition of cell growth under different treatments.

### 4.4. Colony Formation Assay

The cells were cultured in six-well plates in RPMI 1640 medium with 5% (*v*/*v*) FBS at a density of 100 cells per well for 24 h. Cells were then incubated with 0.1% DMSO or indicated treatments for seven days. After the treatment, the cells were stained with 0.4% (*w*/*v*) crystal violet for 10 min. Surplus dye was removed by PBS washing. The plates were air-dried and the images of stained colony formation left on the plates were scanned. The colony formation stained with crystal violet was then solubilized by 0.05 M sodium citrate dissolved in ethanol, and the absorbance of the solution was measured at a wavelength of 595 nm to examine inhibition of colony formation.

### 4.5. Cell Proliferation Assay with CFSE Staining

The cells were cultured in six-well plates in RPMI 1640 medium with 5% (*v*/*v*) FBS at a density of 50,000 cells per well and stained with CFSE for 48 h. Cells were then incubated with 0.1% DMSO or compound treatment for another 24 or 48 h. After the treatment, cell proliferation was detected by FACSan FL1 channel (Becton Dickinson, San Jose, CA, USA). Proliferation index was analyzed with software ModFit LT^TM^ 3.3 (Verity Software House, Topsham, ME, USA) to quantify the inhibition of cell proliferation.

### 4.6. Cell Distribution Analysis with PI Staining

Cells incubated in RPMI 1640 medium with the indicated treatments were harvested by trypsinization, fixed with 70% (*v*/*v*) alcohol at –20 °C for 30 min and washed with PBS. After centrifugation, cells were resuspended with 0.2 mL PI solution which was composed of 0.1% (*v*/*v*) Triton X-100, 100 µg/mL RNase, and 80 mg/mL PI. Cell cycle distribution was detected with FACScan FL2 channel (Becton Dickinson, San Jose, CA, USA) and analyzed with BD CellQuest^TM^ Pro Software.

### 4.7. Measurement of Mitochondrial Membrane Potential Loss with JC-1 Staining

The cells were cultured in six-well plates in RPMI 1640 medium with 5% (*v*/*v*) FBS at a density of 160,000 cells per well for 24 h and then treated as indicated for further 24 h. Cells were incubated in RPMI with 5 µM JC-1 for 10 min at 37 °C and collected through trypsinization. Mitochondrial membrane potential was detected by FACSan FL1 and FL2 channels (Becton Dickinson, San Jose, CA, USA) and analyzed with BD CellQuest^TM^ Pro Software. The loss of mitochondrial membrane potential was observed through detecting R1 (J-aggregates form of JC-1) and R2 (monomeric form of JC-1) cell population by FACScan flow cytometer.

### 4.8. Western Blot Analysis

After treatment, cells were harvested with trypsinization, centrifuged, and lysed in 0.1 mL of lysis buffer containing 10 mM Tris-HCl (pH 7.4), 150 mM NaCl, 1mM EGTA, 1 % Triton X-100, 1 mM PMSF, 10 µg/mL leupeptin, 10 µg/mL aprotinin, 50 mM NaF, and 100 µM sodium orthovanadate. Total protein was quantified, mixed with sample buffer, and boiled at 90 °C for 5 min. Equal amount of protein (30 µg) was separated by electrophoresis in 8% or 12% SDS-PAGE, transferred to PVDF membranes, and was detected with specific antibodies (1:500–1:3000 dilution). The immunoreactive proteins after incubation with appropriately labeled secondary antibody (1:3000 dilution) were detected with an enhanced chemiluminescence detection kit (Amersham, Buckinghamshire, UK).

### 4.9. Confocal Immunofluorescence Microscopy

The cells were seeded on coverslips placed in six-well plates with RPMI 1640 medium with 5% (*v*/*v*) FBS at a density of 110,000 cells per well. Cells were then incubated with indicated treatments. Then, the cells were washed with PBS and fixed with 100% methanol for 15 min. Fixed cells were washed with PBS again, followed by a 0.5-h treatment of 0.1% Triton X-100 to induce membrane permeabilization and 1% BSA in PBS as the blocking solution. The cells were stained with anti-cyclin D1 or anti-cyclin E antibody (1:200 dilutions) for 40 min, followed with fluorescein isothiocyanate (FITC)-conjugated secondary antibody (1:200 dilutions) for 1 h. After washing, 0.15 µg/mL 4′,6-diamidino-2-phenylindole (DAPI) was added for nuclear staining for 5 min. The fluorescence was observed using Confocal microscope Zeiss LSM 880 (Carl Zeiss, Jena, Germany).

### 4.10. Transfection

The cells were cultured in six-well plates in RPMI 1640 medium with 5% (*v*/*v*) FBS at a density of 230,000 cells per well for 24 h. Aliquots containing control plasmid or Myr-Akt expression vector in serum-free Opti-MEM were transfected into Lipofectamine 2000 (Invitrogen, CA, USA) following a 20-min gentle mixing at room temperature. Cells were incubated in Opti-MEM containing mixtures (plasmid concentration: 2 µg/µL) for 6 h at 37 °C. After the incubation, cells were washed with medium and incubated in 10% FBS-containing RPMI-1640 medium for 48 h, treated with indicated treatments, and collected to perform the Western blot analysis.

### 4.11. Measurement of Reactive Oxygen Species (ROS) Production

The cells were cultured in 12-well plates in RPMI 1640 medium with 5% (*v*/*v*) FBS at a density of 100,000 cells per well for 24 h. Cells were then treated with 0.1% DMSO or compound. For the measurement of ROS production, cells were incubated with 10 µM DCF-DA for 30 min at 37 °C. ROS production was detected by FACSan FL1 channel (Becton Dickinson, San Jose, CA, USA) and analyzed with BD CellQuest^TM^ Pro Software.

### 4.12. Measurement of Intracellular Ca^2+^ Content

The cells were cultured in 12-well plates in RPMI 1640 medium with 5% (*v*/*v*) FBS at a density of 100,000 cells per well for 24 h. Cells were then treated with 0.1% DMSO or compound. For the measurement of intracellular Ca^2+^, the cells were incubated with 5 µM fluo-3 AM (acetoxymethyl ester) for 30 min at 37 °C. Intracellular Ca^2+^ was detected by FACSan FL1 channel (Becton Dickinson, San Jose, CA, USA) and analyzed with BD CellQuest^TM^ Pro Software.

### 4.13. Data Analysis

Data are expressed as mean ± SEM of at least three independent experiments. Computerized image analysis system Lab^TM^ Software (Bio-Rad Laboratories, Hercules, CA, USA) was adopted to quantify experimental results of Western blot analysis. Statistical analysis of data for multiple groups was performed by a Student’s *t*-test and *p*-values less than 0.05 were considered statistically significant.

## Supplementary Materials

The following are available online. Figure S1: Effect of (+)-Brevipolide H on colony formation in PC-3 cells, Figure S2: Effect of (+)-Brevipolide H on activation of caspases in PC-3 cells, Figure S3: Effect of NAC on (+)-Brevipolide H-mediated cell cycle distribution in PC-3 cells, Figure S4: Effect of (+)-Brevipolide H on Ca^2+^ flux in PC-3 cells.

## Abbreviations

CFSE	carboxyfluoresceinsuccinimidyl ester
CRPC	castration-resistant prostate cancer
DCF-DA	2′,7′-dichlorodihydrofluorescein diacetate
DTT	DL-dithiothreitol
ER	endoplasmic reticulum
InsP3	inositol trisphosphate
MAPK	mitogen-activated protein kinase
mTOR	mammalian target of rapamycin
Myr-Akt	myristoylated Akt
NAC	N-acetyl-L-cysteine
PI3K	phosphoinositide 3-kinase
PI	propidium iodide
PMSF	phenylmethanesulfonyl fluoride
PTEN	phosphatase and tensin homologue
ROS	reactive oxygen species
SERCA	sarco/endoplasmic reticulum Ca^2+^-ATPase
SRB	sulforhodamine B
TCA	trichloroacetic acid
