# Supplementary figures and images for: The (+)-Brevipolide H Displays Anticancer Activity against Human Castration-Resistant Prostate Cancer: The Role of Oxidative Stress and Akt/mTOR/p70S6K-Dependent Pathways in G1 Checkpoint Arrest and Apoptosis
Source: Molecules. 2020 Jun 25;25(12):2929. doi: 10.3390/molecules25122929 (PMC7355498; doi:10.3390/molecules25122929)

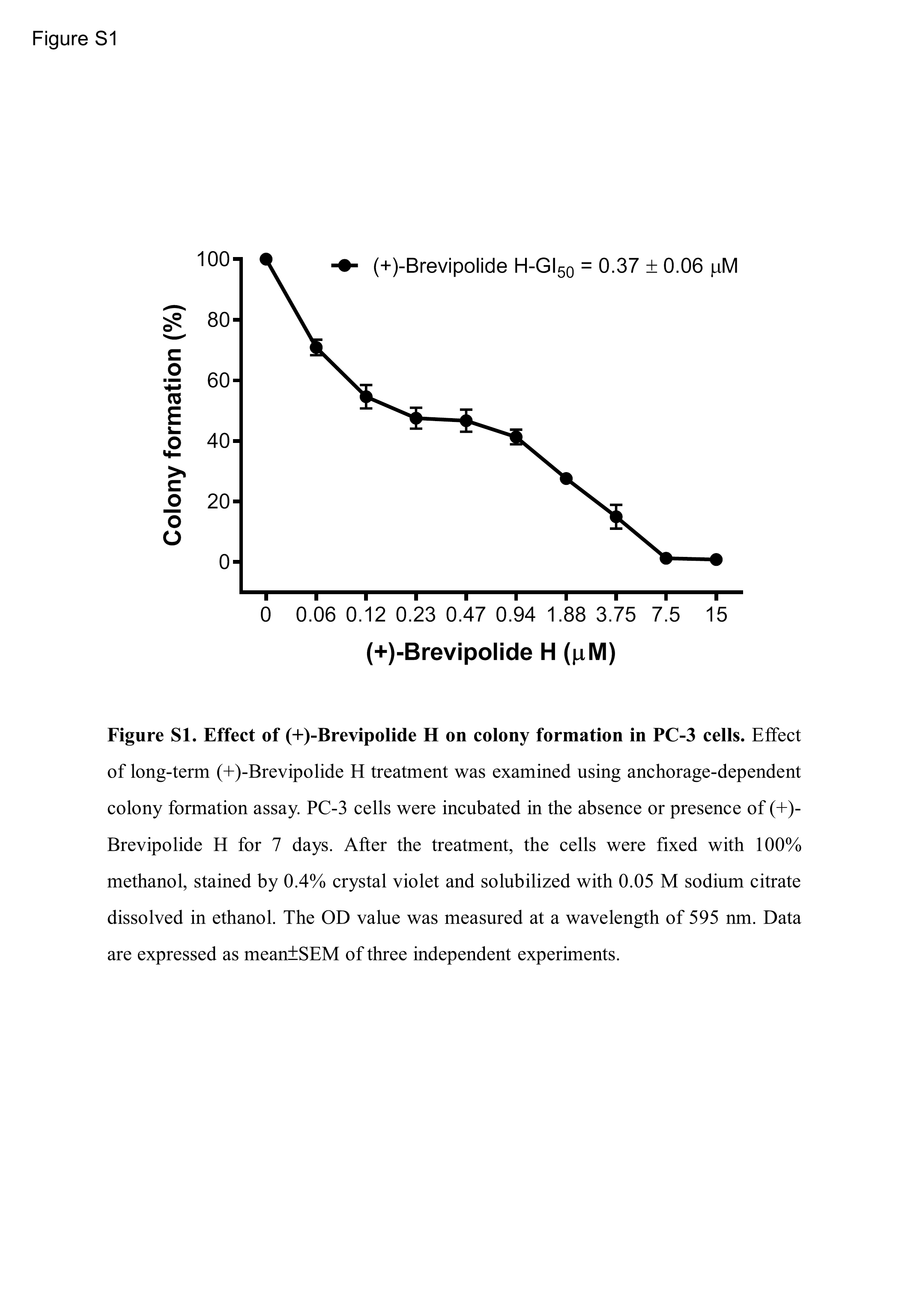

Supplement: Supplementary file 1 [file molecules-25-02929-s001.zip › Supplementary figures/Figure S1.tif]

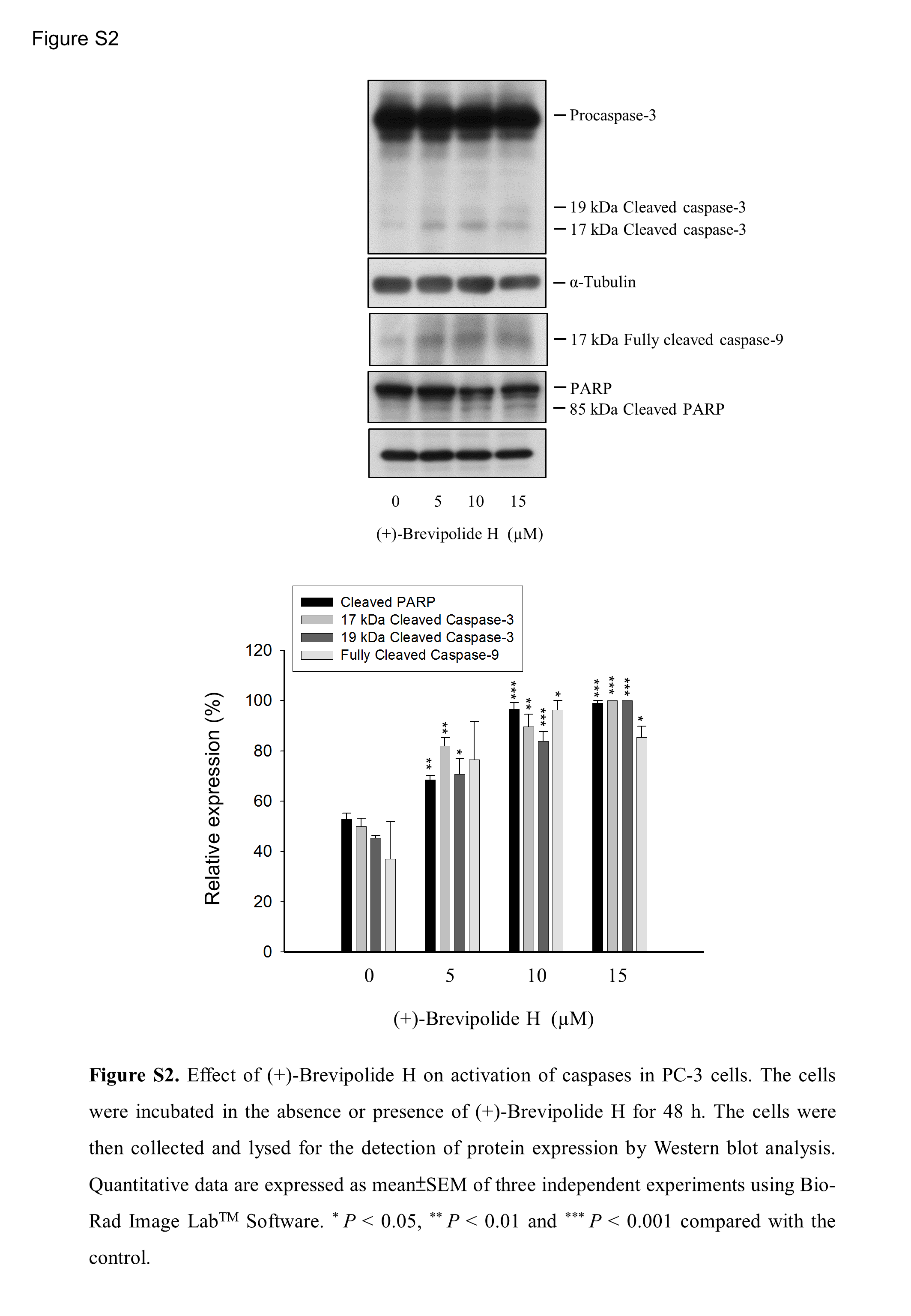

Supplement: Supplementary file 1 [file molecules-25-02929-s001.zip › Supplementary figures/Figure S2.tif]

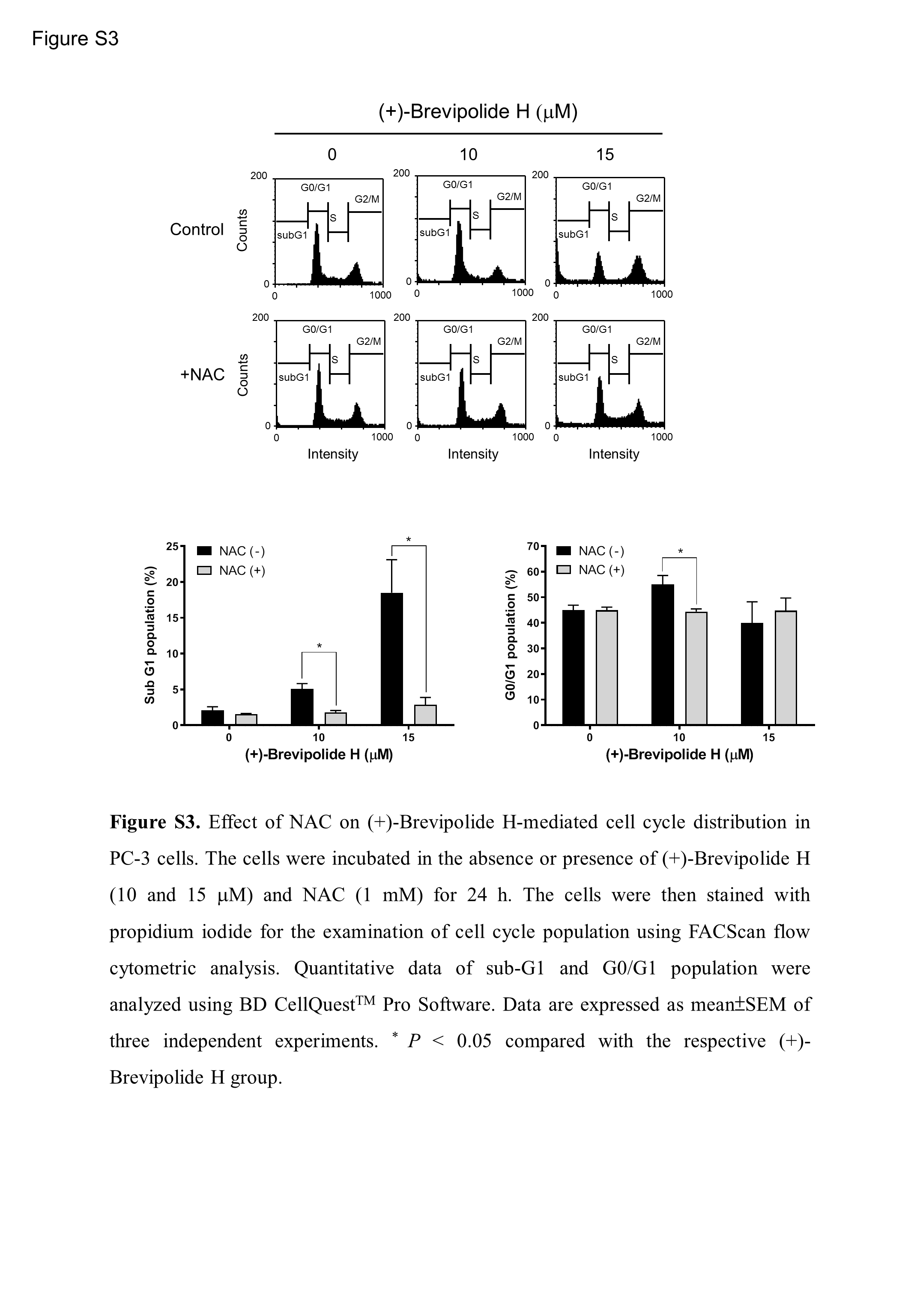

Supplement: Supplementary file 1 [file molecules-25-02929-s001.zip › Supplementary figures/Figure S3.tif]

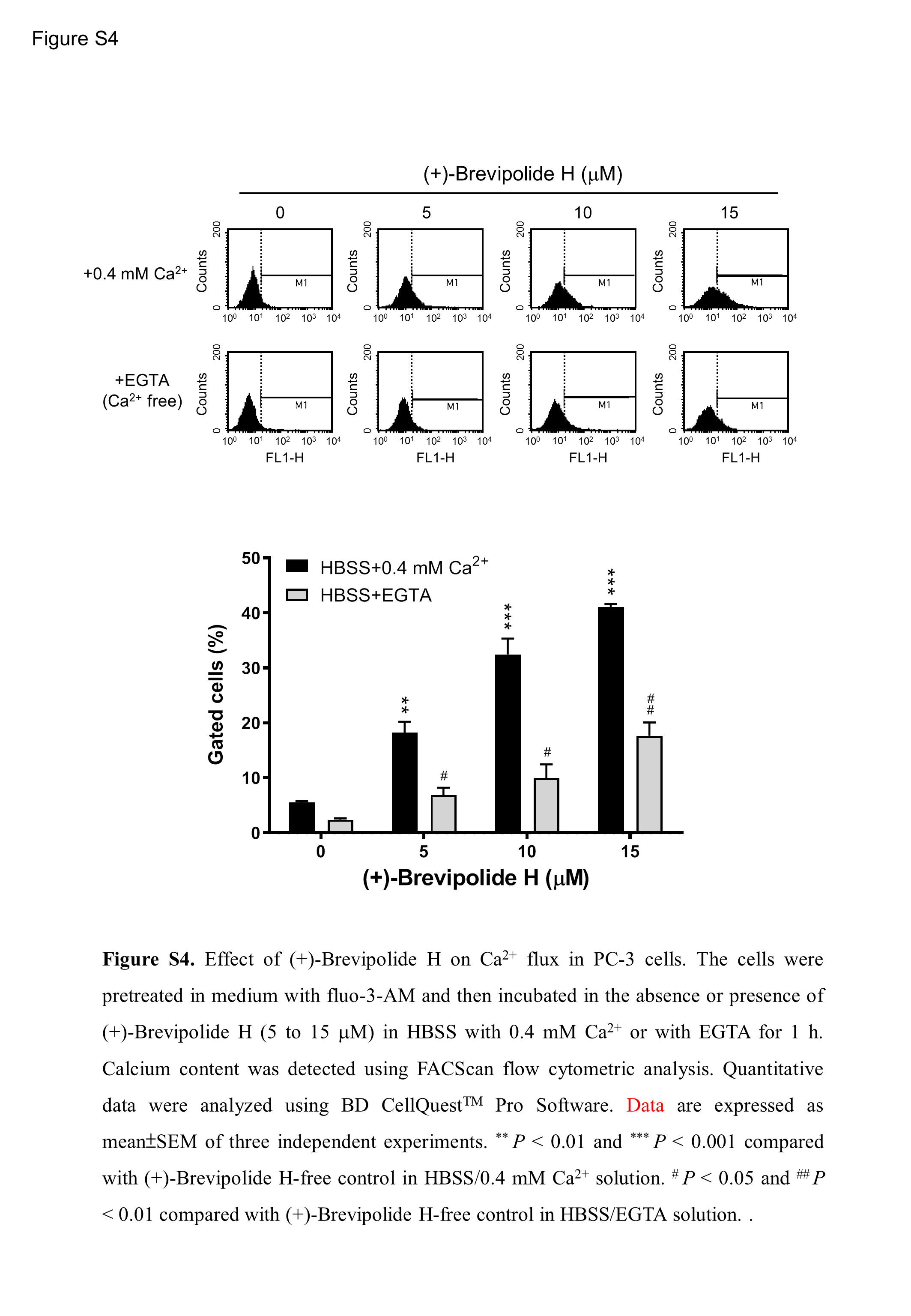

Supplement: Supplementary file 1 [file molecules-25-02929-s001.zip › Supplementary figures/Figure S4.tif]
